# Swept-source optical coherence tomography changes and visual acuity among Palestinian retinitis Pigmentosa patients: a cross-sectional study

**DOI:** 10.1186/s12886-021-02047-6

**Published:** 2021-07-29

**Authors:** Orjowan Shalabi, Zaher Nazzal, Muath Natsheh, Salam Iriqat, Michel Michaelides, Muyassar Ghanem, Alice Aslanian, Yahya Alswaiti, Alaa AlTalbishi

**Affiliations:** 1 St. John of Jerusalem Eye Hospital Group, East Jerusalem, 91198 Palestine; 2 Department of Family and Community Medicine, Faculty of Medicine and Health Sciences, An-Najah National University, Nablus, Palestine; 3 Department of genetics, Moorfields Eye Hospital and UCL Institute of Ophthalmology, London, UK

**Keywords:** Retinitis Pigmentosa, SS-OCT, Ellipsoid zone, Cystoid macular edema, Visual acuity

## Abstract

**Background:**

Retinitis pigmentosa (RP) is a heterogeneous group of inherited ocular diseases that result in progressive retinal degeneration. This study aims to describe different Swept-source Optical Coherence Tomographic (SS-OCT) changes in Palestinian RP patients and to explore possible correlations with Visual Acuity (VA).

**Methods:**

A cross-sectional observational study was conducted on Retinitis Pigmentosa patients diagnosed with RP in a tertiary eye hospital. Full history and ocular examination were made. SS-OCT imaging was done for all eyes assessing the presence of cystoid macular edema, epiretinal membrane, macular holes, and external limiting membrane, ellipsoid zone status. Also, central macular thickness and choroidal vascular thickness were measured.

**Results:**

The study was run on 161 eyes of 81 patients; 53 males and 28 females. The average age at examination was 26.1 (6–78) years. Twenty-six eyes (16.1%) were of syndromic RP patients, mostly Usher syndrome; 20 eyes (12.4%). The mean Logaritmic minimal angle of resolution (LogMAR) of Best Corrected Visual Acuity (BCVA)of the study sample was 0.66 ± 0.7. The most prevalent change was cystoid macular edema [28 eyes, (17.4%)], followed by epiretinal membrane [17eye, (10.6%)]. A macular hole was noted only in one eye (0.6%). Ellipsoid zone and external limiting membrane were absent in 55 eyes (35.0%) and 60 eyes 37.5%. Vitreous hyperreflective foci were found in 35 eyes (43.8%). LogMAR of BCVA was associated significantly with cystoid macular edema (*p* = 0.001), ellipsoid zone(*p* = 0.001), and external limiting membrane (*p* = 0.001).

**Conclusions:**

Detailed SS-OCT assessment in Palestinian patients diagnosed with RP identified different morphologies from other populations. Cystoid macular edema and vitreous hyperreflective foci may reflect signs of early or intermediate stages of the disease. Disease progression can be monitored by measuring the length/width (area) of ellipsoid zone +/− external limiting membrane and choroidal vascular thickness, which should be evaluated serially using high-resolution OCT.

## Introduction

Retinitis pigmentosa (RP) is a clinically and genetically heterogeneous group of hereditary retinal disorders [1], comprising the most common inherited retinal degeneration, affecting around 1 in 2–3000 individuals [2].

More than 100 genes have been associated with RP [3]. Disease-causing variants in these genes cause progressive loss of rod photoreceptor function, followed cone function, often leading to complete blindness [4]. Thus, patients with RP first suffer from peripheral, then central visual loss. Central visual loss may also be attributed to cystoid macular edema (CME), which can be treated by different modalities, including oral and topical carbonic anhydrase inhibitors, steroids, and Aflibercept [5], or may be attributed to posterior subcapsular cataract [6].

Assessment of patients with RP includes a detailed history, full standard ophthalmological exam, imaging modalities, and functional testing, with molecular genetic testing increasingly being undertaken [7].

Over the last two decades, the study of retinal anatomy and pathology has been revolutionized by the advent of optical coherence tomography (OCT) [8]. This began with time-domain OCT in 1991. By 2006, spectral-domain OCT had dramatically improved image resolution, motion artifact, and acquisition time. However, imaging of subretinal structures could be acquired using Enhanced Depth Imaging software at the expense of higher retinal structures [9].

Swept-source (SS) OCT is a variation of OCT that offers improvements in visualizing the vitreous, retina, and choroid at one image. The increased scan speeds, decreased signal attenuation, more comprehensive imaging, and deeper tissue penetration make SS-OCT ideal for capturing a wide range of views and studying structures below the retinal pigmented epithelium (RPE), especially the choroid [9].

OCT can show progressive degeneration of the photoreceptor layers through the change in the integrity of the outer retinal hyperreflective bands [10]. OCT also enables the detection of macular abnormalities like CME, epiretinal membrane (ERM), vitreomacular traction interface abnormalities, or macular holes [11, 12]. However, few studies have been conducted to evaluate the association between visual acuity (VA) and OCT changes. Previous studies have shown that the ellipsoid zone (EZ) was associated with better VA and thicker fovea in RP patients. At the same time, the absence of EZ may reflect foveal dysfunction in patients with RP [12].

The Palestinian community has a relatively high consanguinity rate, which significantly increases the prevalence of recessively inherited disorders such as RP [13, 14]. A greater understanding of disease patterns, characteristics, and factors affecting visual function will help monitor disease progression and evaluate patients who may benefit from treatment trials and cataract surgery. This study aims to describe a range of SS-OCT changes in Palestinian patients with RP and to explore any correlation between these retinal disturbances and best-corrected visual acuity (BCVA).

## Materials and methods

A cross-sectional observational study was conducted on all patients diagnosed with RP at St. John Eye Hospital between October 2016 and December 2018. Informed consent was obtained from all participants and from parents and/or legal guardians of participants under 18 years of age. Examinations were carried out in accordance with the Code of Ethics of the World Medical Association (Declaration of Helsinki) and to ensure the privacy and confidentiality of the participants. Approval of the St John Eye Hospital institutional ethics committee was obtained.

The study included 161 eyes of 81 patients diagnosed with RP. Diagnosis of RP was based on typical clinical history, fundoscopic appearance, fundus autofluorescence, and full-field ERG. The study included patients of all age groups, all modes of inheritance, and both syndromic and non-syndromic RP. We excluded patients with any other retinal pathology or systemic disease that might cause macular changes on OCT. Excluded comorbidities included diabetic retinopathy, central serous Chorioretinopathy, age related macular degeneration, retinal vein occlusion, Stargardt disease and cone-rod dystrophy. Additionally, patients with media opacity precluding OCT image acquisition and patients with poor image quality were excluded.

A total of 100 patients with RP registered in the hospital’s database were recruited for a single visit, of them, 19 patients were excluded according to the above- mentioned criteria (patients with other differential diagnosis, non -available OCT images due to young age or non-cooperation of patients). Full history and standard ophthalmological examination were performed. BCVA was measured with the logarithm of the minimal angle resolution (logMAR) vision chart.

OCT imaging was done using the DRI Triton, Swept Source OCT (Topcon Inc., Tokyo, Japan) device by a dedicated imaging technician. SS-OCT imaging was performed using horizontal 9 mm wide raster of 128 scans and 16-line radial 6 mm wide scans. Three readers did SS-OCT image assessment; two retina specialists (TA, SY), and an OCT specialist (ES). The agreement of two readers, out of three, decided the final decision. Image assessment and measurements were done with the aid of IMAGE net 6 OCT Topcon software.

**Fig. 1:**
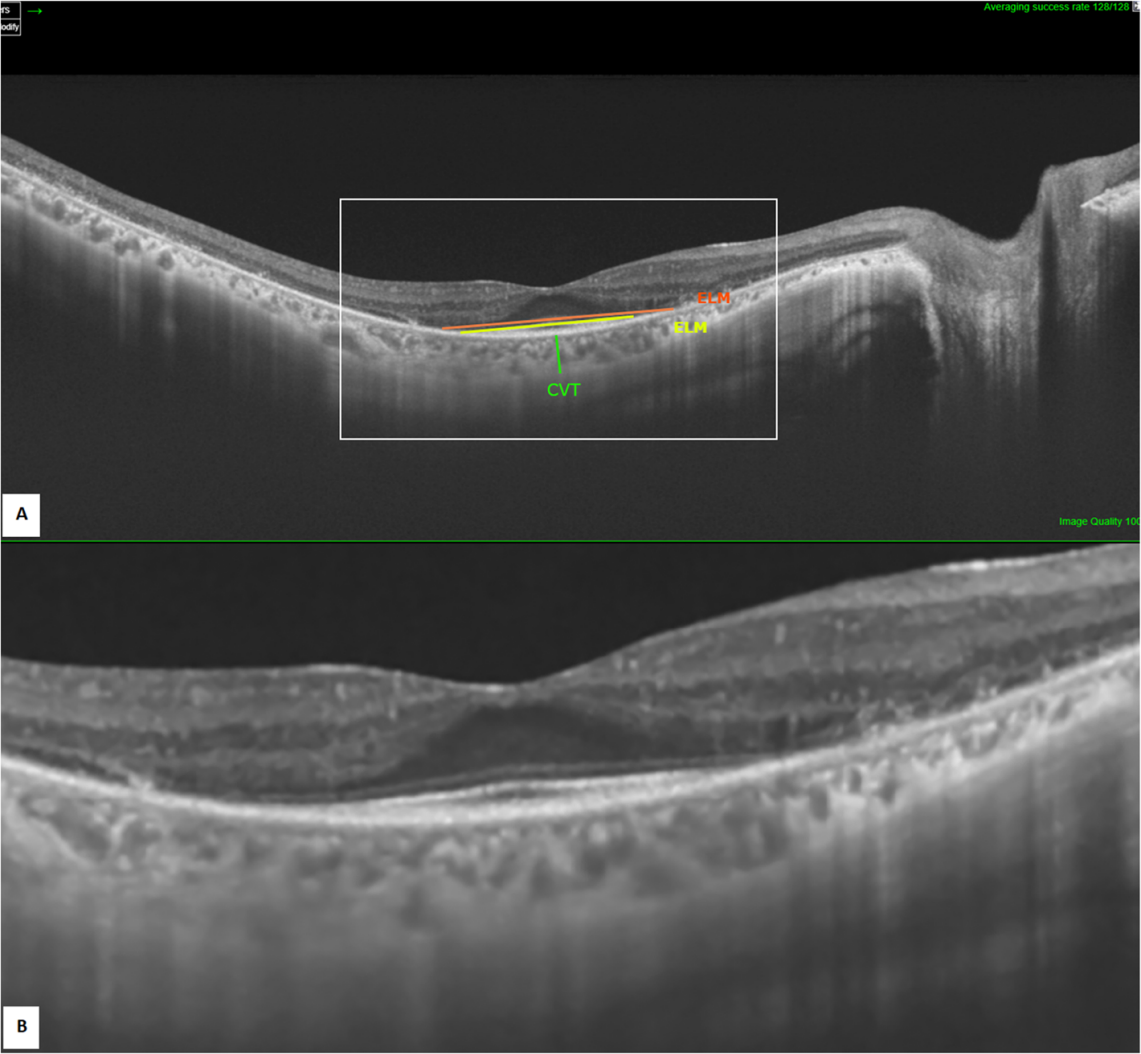
SS-OCT image of the right eye (**A**: annotated scan, **B**: magnified unannotated scan) showing measurement of EZ (in yellow), ELM (in orange), and subfoveal CVT (in green)

The OCT variables assessed included; (i) macular changes: presence or absence of CME, macular hole, and automated measurement of Central macular thickness (CMT) in μm; (ii) the presence or absence of other retinal changes: ERM, choroidal neovascularization (CNV), vitreomacular interface abnormalities [15] including vitreomacular traction (VMT) and vitreomacular adhesion (VMA); (iii) EZ status was assessed: EZ width/length was manually measured starting from the last visible point at the temporal side to the last visible point at the nasal side using IMAGEnet 6 OCT software [Fig.1], and then grouped into three groups based on length; normal if more than 1500 μm, abnormal if less than 1500 μm, and absent [16]. A similar assessment was applied for the External Limiting Membrane (ELM) status and grouped into related three groups. Fourth, manual measurement of the subfoveal choroidal vascular thickness (CVT) by taking the vertical distance under the fovea from the RPE’s external edge to the end of the choroido-scleral junction (Fig. 1). Finally, the presence or absence of vitreous hyper-reflective foci (vitreous HRF) was established in patients for whom high-definition images were possible. These variables were compared to relative findings of other studies as to be mentioned in the discussion section.

Descriptive statistics were first applied to assess the patients’ baseline characteristics (mean with standard deviation [SD] for continuous variables, number, and percentage for categorical variables). A Chi-square test was used to compare categorical variables. Independent t-test and Mann-Whitney test were used to compare means in normally distributed data and nonparametric data. Kruskal Wallis test was used for comparing nonparametric values in more than two groups. Possible correlations between continuous variables were explored using the Spearman’s ranked order correlation test. A *P*-value of less than 0.05 was considered to be statistically significant. All statistical analyses were done using SPSS version 20.0.

## Results

This study recruited 81 RP patients and assessed 161 eyes. It included 53 males (65.2% of eyes) and 28 females (34.8% of eyes). The average age at examination was 26.1 (6–78) years. Twenty-six eyes (16.1%) were from patients with syndromic RP, including Usher syndrome (20 eyes, 12.4%), Bardet-Biedl syndrome (4 eyes, 2.5%), and RP with unspecified syndromic features (2 eyes, 1.2%). This sample’s mean LogMAR BCVA was 0.66 ± 0.73 (0.00–3.00), and the mean CMT was 230 ± 85.7 (86–577) μm. The subfoveal CVT thickness could be measured in 72 eyes, with an average subfoveal CVT being 289.3 ± 103.3 (69–612) μm (Table 1).

**Table 1:** Baseline characteristics of the studied Retinitis Pigmentosa cases (*n* = 161 eyes)

Characteristics	Eyes (%)	Mean ± SD ***(Min-Max)***
**Sex**		
Male	105 (65.2%)	
Female	56 (34.8%)	
***Age in years***		26.1 ± 15.3 *(6–78)*
**LogMAR BCVA**		0.66 ± 0.73 *(0.00–3.00)*
**Inheritance**		
Autosomal Recessive	142(88.2%)	
X-linked	2 (1.2%)	
Sporadic	6 (3.7%)	
**Syndromic RP**		
Yes	26 (16.1%)	
No	135 (83.9%)	
**CMT**		230.0 ± 85.64 (86–577)
Subfoveal CVT*		289.3 ± 103.33 (69–612)

*Assessed in 72 eyes

The most prevalent change was CME’s presence (28 eyes, 17.4%); of the Syndromic RP patients, (7 eyes, 26.9%) had CME, compared to (21 eyes, 15.6%) of non-syndromic patients; however, this was not statistically significant (*p* = 0.161). The second most common finding was ERM (17 eyes, 10.6%). A macular hole was noted only in one eye (0.6%) of the studied sample. EZ was reported to be absent in 55 eyes (35.0%) and abnormal in 63 eyes (40.1%). ELM was found to be missing in 60 eyes (37.5%) and abnormal in 45 eyes (28.1%). However, no cases of VMT/VMA or CNV were observed (Table 2).

**Table 2:** Prevalence of SS-OCT changes of the studied Retinitis Pigmentosa cases (161 eyes)

SS-OCT changes	Eyes (%)
**Cystoid macular edema (CME)**	
Present	28 (17.4%)
Absent	133 (82.6%)
**Epiretinal membrane (ERM)**	
Present	17 (10.6%)
Absent	144 (89.4%)
**Macular Hole**	
Present	1 (0.06%)
Absent	160 (99.4%)
**Ellipsoid Zone (EZ)**	
Intact (Normal)	39 (24.8%)
Absent	55 (35.0%)
Abnormal	63 (40.1%)
**External Limiting Membrane (ELM)**	
Intact	55 (34.4%)
Absent	60 (37.5%)
Abnormal	45 (28.1%)
Vitreal Hyper-reflective foci (HRF)*	
Present	35 (43.8%)
Absent	45 (56.2%)

*Assessed in 72eyes

Vitreous Hyper-reflective foci (HRF) were examined in 72 eyes and were found in 35 eyes (43.8%) of the cases assessed. Of these (with foci present), 26% were associated with CME in a non-significant relationship (*p* = 0.260), and 17.1, 60% had an absent, abnormal EZ respectively in a non-significant relationship (*p* = 0.197).

Table 3 presents the baseline and clinical characteristics, including BCVA. It was noted that there was a significant association between sex and BCVA (*p* = 0.007), where females had worse VA than males. CME had a highly significant relationship with BCVA (*p* < 0.001); interestingly, eyes without CME had a much worse VA in terms of BCVA. Both the EZ and ELM status shows a highly significant relationship with BCVA (*p* < 0.001), with worse VA associated with EZ and/or ELM being absent. At the same time, the presence of ERM does not show a significant relationship with BCVA. Similarly, the presence of vitreous HRF had no significant association with BCVA.

**Table 3:** Association of BCVA with Baseline and clinical characteristics patients

Variable	LogMAR BCVA Mean (±SD)	*P*-value
**Sex**		
Male	0.545 (±0.64)	0.007^*^
Female	0.873 (±0.84)	
**RP associated with a syndrome**		
Yes	0.667(±0.67)	0.340^℗^
No	0.661 (±0.75)	
**CME**		
Present	0.33(±0.22)	< 0.001^*^
Absent	0.73(±0.79)	
**Ellipsoid Zone**		
Intact	0.430(±0.55)	
Abnormal	0.408(±0.35)	< 0.001^**^
Absent	1.13(±0.97)	
**External Limiting Membrane**		
Intact	0.415(±0.47)	
Absent	1.11(±0.93)	< 0.001^**^
Abnormal	0.407(±0.39)	
**ERM**		
Present	0.402 (±0.69)	0.125^℗^
Absent	0.693 (±0.6)	
**Vitreous hyperreflective foci**		
Present	0.590 (±0.66)	0.551^℗^
Absent	0.684 (±0.69)	

^**^Independent-Samples Kruskal Wallis test, ^℗^Mann Whitney U test, *Independent t-test

Spearman’s ranked order correlation test was used to assess the correlation between BCVA and CMT, CVA and age. We observed a weak significant negative correlation between CMT and BCVA (r = − 0.224, *p* = 0.005) and between subfoveal CVT and BCVA (r = − 0.260, *p* = 0.015). Additionally, subfoveal CVT was found to be negatively correlated with age (r = − 0.23, *p* = 0.039). We repeated the analysis using data of one eye that was randomly selected from patients with two eyes. The results showed a non-significant week negative correlation between BCVA and CMT (r = − 0.124, *p* = 0.278), sub foveal CVT (r = − 0.173, *p* = 0.273), and age (r = − 0.106, *p* = 0.693).

## Discussion

Studying retinal changes has been revolutionized with the advent of OCT, especially spectral-domain OCT. Recently, the new emerging SS-OCT is being assessed clinically and may add new insights to the current understanding of retinal diseases.

In this study, the average age at examination is 26.1 (6–78); younger than those reported in other studies; 33.4–49.3 years [17, 18]. This can be explained by higher prevalence of inherited disease in the Palestinian population due to higher consanguinity rates; reaching up to 45%, according to the Palestinian Central Bureau of Statistics, which are considered to be among the highest in the Middle East region.

. A wide range of CME prevalence in RP patients has been reported in previous studies, where some reported as small as 5.5% by Hagiwara [18], Adackapara [11] has reported 47%, using time-domain OCT, compared to a 17.4% prevalence of CME in our cohort. However, researchers using the spectral domain OCT identified a variety of CME prevalence; 50.9% by Liew et al. [19], 25.9% by Kim et al. [16], 12.5% by Triolo et al. [18], and 14.5% by Chebil et al. [20]. This large variance in CME prevalence can be due to the range of sample size and baseline characteristics and the OCT device’s sensitivity. Nevertheless, CME is still the most frequent OCT change in RP patients [21].. ERM (10.6%) was the second most frequent change noted. This is in line with the findings of Chebil et al. [20], which documented EMR in 8.2% of RP patients. Others reported varying proportions, ranging from 0.6 to 28% [18, 19, 22–24]. Overall, ERM represented the second most frequent change noted in these studies. We found that ERM had no significant correlation with VA. A similar finding was reported by Ibrahim et al. [24]; However, when ERM is studied as part of Vitreomacular Interface Disorders (VMIAs), the prevalence was over 40% of the eyes, but its direct association with VA was not examined [16].

Macular holes were found less frequently, with only one eye (0.6%) displaying a lamellar hole, similar to those recorded by Hagiwara et al. (0.5%) [23]. Previous studies reported a higher prevalence of macular holes [22, 24]. Employing the American Association of ophthalmology classification, no cases of VMT were encountered in this study, whereas 5 of 622 eyes (0.8%) and 58 of 1161 eyes (5%) with VMT reported in other studies [23, 25]. Similarly, no cases of CNV have been identified in this study, as also noted by Ibrahim et al. [24], whereas 1.7% of eyes with CNV have been reported by Triolo et al. [18]

This study identified a higher percentage of eyes having an absent EZ (35%) compared to other studies [18, 25–27]. We found a highly significant correlation between EZ status and BCVA, as describes in other studies [16, 24, 27–29].. The improvement in SS-OCT’ s ability to delineate EZ from other hyper reflexive OCT lines as opposed to other OCT systems for other studies can also explain this. Absent ELM was observed in 37.5% of eyes, much higher than reported others, 13.6% by Triolo et al. [18], and 25% by Battaglia et al. [27] using spectral-domain OCT. This may be explained by the more severe disease status of our sample, but also, it may relate to the higher image resolution of SS-OCT. The degeneration of the ELM was also shown to be highly correlated with BCVA (*P* < 0.001). Indeed, Battaglia et al. showed that ELM remains significantly associated with BCVA after multivariate regression, whereas EZ did not [27].

In this study, BCVA was found to be significantly correlated with several variables. The first is gender; females, in this study, had a worse BCVA than males. (0.873 vs. 0.545, *p* = 0.007). This may be related to the cultural beliefs of the studied population, and females are less likely to seek medical attention at an earlier stage [30]. The second is CME, with a highly significant correlation with BCVA (*p* < 0.001). This contrasts with the non-significant relationship reported by others [16, 23, 24, 27]. It may relate to the noteworthy finding in this study, that eyes without CME had a worse BCVA than eyes with CME (LogMAR 0.73 vs. 0.33). This may indicate that the non-CME eyes may be atrophic, thereby eyes with more advanced disease status rather than an earlier stage, at which stage CME is not a manifestation.

In this study, the average CMT for RP patients was marginally above the healthy population (230.0 ± 85.649 μm) and correlated with decreased BCVA (*p* = 0.005) [31].The average CMT decreases to 210 ± 63.1 μm when eyes with CME are excluded, leading to worse visual outcomes in terms of (LogMAR =0.73 ± 0.79). This is likely due to CME being indicative of earlier disease status and thereby better BCVA. A comparison between CMT of CME & non-CME eyes was also made by Kim et al., who found no significant difference in BCVA between groups [16]. A significant relationship between CMT (avg of 180 μm) and BCVA was observed by Ibrahim et al. [24]. This suggests a bidirectional relationship between CMT and vision; both thickened and atrophic maculae showed decreased vision.

It has been hypothesized that the factor involved in photoreceptor degeneration is choroidal thinning caused by decreased blood flow [32]. Our study showed that the subfoveal CVT was 289.3 ± 103.3, almost similar to others’ findings [33–35], but still lower than the normal range. This correlates significantly with age (*p* = 0.039), as expected, and negatively with BCVA (*p* = 0.015). Similar significant relationships were noted by Aknin et al. [33] and Ayton et al. [34] but inconsistent with the results of Dhoot et al. (33)and Sodi Et al. [36] using Enhanced Depth Imaging OCT. This supports the observation that the greater the choroidal thinning at the subfoveal area, the worse the BCVA.

HRF has been identified in other retinal diseases composed of macrophages, migrating RPE cells, and extravasated lipoprotein. This study is the first to describe HRF in the vitreous, thereby increasing our limited current understanding of their role in the disease process. Although vitreous HRF was present in 43.8% of studied eyes, this was non-significantly correlated with BCVA (*p* = 0.551). Interestingly, eyes without vitreous HRF had a worse BCVA than eyes with HRF (LogMAR 0.684 vs. LogMAR 0.590). This may indicate that their presence reflects an early or intermediate stage of the disease, rather than an advanced stage. This is also supported by the fact that they were more likely to be found when the EZ was shortened and abnormal (60%) rather than when absent. To date, HRF has been studied in other retinal layers, but not in the vitreous. And vitreous HRF was only studied in diabetic retinopathy and uveitis.

Although previous articles in the literature have studied retinal changes in patients with RP and their correlation with BCVA, this study is one of the first to do so using SS-OCT. Moreover, this is perhaps the most comprehensive study involving the assessment of changes in the vitreous, retina, and choroid, including vitreous HRF being studied for the first time. However, this study has some limitations that should be taken into consideration when interpreting its findings. First limitation is the lack of genetic diagnosis in these patients, and so no genotype-phenotype correlation could be explored. Another limitation is that the generalizability is limited to other populations of high rates of consanguinity similar to the Palestinian population.

## Conclusions

Detailed SS-OCT assessment in Palestinian patients diagnosed with RP identified different morphologies from studies of other populations, which may, in part, be related to the as yet uncharacterized genetic causes of RP in Palestine, as well as the younger age of presentation. CME and vitreous HRF may be considered signs associated with early or intermediate stages of the disease.

Disease progression can be monitored by measuring the length/width (area) of EZ +/− ELM and CVT, which should be evaluated using high-resolution OCT better to identify candidates for gene therapy trials/treatments; as well as identify CME.

## Data Availability

The datasets generated during and analyzed during the current study are not publicly available due to ongoing other researches but are available from the corresponding author on reasonable request.

## Abbreviations

BCVA: Best-corrected visual acuity
CME: Cystoid macular edema
CMT: Central macular thickness
CNV: Choroidal neovascularization
CVT: Choroidal vascular thickness
ELM: External limiting membrane
ERM: Epiretinal membrane
EZ: Ellipsoid zone
HRF: Hyper-reflective foci
LogMAR: Logarithm of minimal angle of resolution
OCT: Optical coherence tomography
SS-OCT: Swept-source OCT
RP: Retinitis pigmentosa
RPE: Retinal Pigmented Epithilium
VA: Visual acuity
VMA: Vitreomacular adhesion
VMT: Vitreomacular traction
